# Use heat with caution! Pleurobiliary fistula after hepatocellular carcinoma microwave ablation in lymphoma patient: A case study

**DOI:** 10.1016/j.radcr.2022.07.102

**Published:** 2022-08-10

**Authors:** Etedal Abdurabuh, Mutaz Khairo, Aquib Bakhsh, May Alsharif, Wael AlYamani

**Affiliations:** a Department of Radiology, Al Noor Specialist Hospital, Makkah, Saudi Arabia; b Department of Radiology, King Abdullah Medical City (KAMC), Makkah, Saudi Arabia

**Keywords:** Pleurobiliary fistula, Hepatocellular carcinoma, Microwave ablation, Lymphoma

## Abstract

Thermal ablation by microwave ablation (MWA) or radiofrequency ablation (RFA) is a frequently used technique for hepatic lesion treatment due to its low rate of complications. Surgery, transarterial chemoembolization (TACE), and yttrium-90 (Y-90) transarterial radioembolization (TARE) are other ways to treat hepatic lesions. Thoracobiliary fistula (TBF) is a rare side effect of thermal ablation. Other side effects include vascular injury, damage to the biliary system, and infection. We present the case of a 62-year-old male patient who has a history of lymphoma and was diagnosed with a hepaticellular carcinoma lesion on follow-up CT in segment VII, which appeared in close relation to the right diaphragm. The patient had been through several interventional procedures, including Y-90 therapy, TACE, and MWA with thermal impact, which resulted in a biloma forming and ramping up the progression of pleurobiliary fistula, which is confirmed by HIDA scan, this case highlights the significance of monitoring patients after thermal ablation, particularly in cases of large justa-diaphragmatic tumors, to detect any diaphragmatic or biliary tree injuries.

## Introduction

When it comes to the treatment of hepatic lesions, surgical resection can be considered as the most effective approach. However, other treatment options, such as radiofrequency and microwave ablation (MWA), transarterial chemoembolization (TACE), and yttrium-90 (Y-90) trans-arterial radioembolization (TARE), have recently been shown equal effectiveness as surgical resection in small, medium, and large hepatocellular carcinoma (HCC). Although the thermal ablation is minimally invasive and relatively low risk, complications can occur [1]. Thoracobiliary fistula (TBF) is a rare side effect of ablation in which a connection is made between the biliary system and either the pleura or the bronchial tree. Relatively few published papers have reported on TBF after liver cancer ablation. Here, we report a case of TBF in a patient with hepatocellular cancer following microwave ablation.

## Case report

**Fig. 1:**
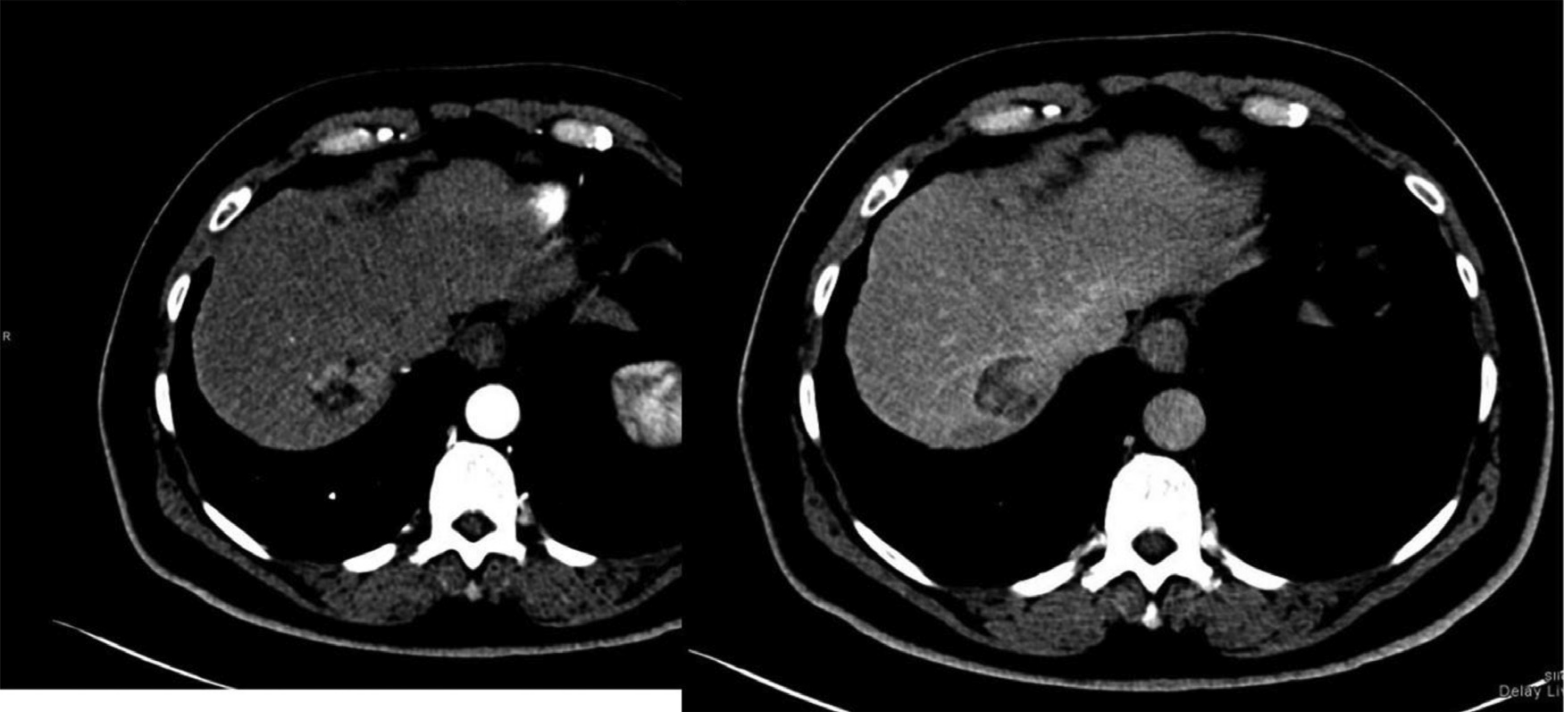
Segment VII liver HCC. Abdominal enhanced CT axial images in the arterial (A) and delayed phases (B). A 3.7-cm segment VII liver lesion with arterial enhancements and delayed washout with capsular enhancement compatible with HCC.

We describe the case of a 62-year-old Saudi male patient with chronic hepatitis B virus infection on entecavir prophylaxis who was diagnosed with Stage IA peripheral T-cell lymphoma in December 2017. He completed 6 cycles of CHOP medications in April 2018 with no complications. During follow-up, his abdominal computerized tomography (CT) scan revealed a 5.5-cm hypodense lesion with a cirrhotic liver appearance (Fig. 1). The lesion was seen in segment VII, compressing the inferior vena cava (IVC) and right hepatic vein. It showed a typical HCC appearance and was labelled as Liver Imaging Reporting and Data System-5 (LI-RADS-5).

At the same time, his blood work showed an aspartate transaminase (AST) level of 21 U/L (reference, 8-48 U/L), an alanine transferase (ALT) level of 36 IU/L (reference, 12-78 U/L), a total bilirubin level of 0.53 mg/dL (reference, <1.2 mg/dL), alkaline phosphate 133 U/L (reference, 45-115 U/L), an albumin level of 3.56 gm/dl mL (reference, 3.4-5 gm/dL), an alpha-fetoprotein level of 5 ng/mL (reference, 0-8.1 ng/mL), CEA level of 3.46 ng/mL (reference, 0-2.5 ng/mL), and a carbohydrate antigen (CA) 19-9 level of 16.58 U/mL (reference, 0-30.9 U/mL).

In September 2020, he was treated for an HCC lesion with TARE using Y-90. A pretreatment injection of a Tc-99m MAA radiotracer showed that there was no significant shunting outside of the liver. Under the guidance of ultrasound, an injection of 1 GBq of Y-90 SIR-spheres by Sirtex (resin microspheres) from the right hepatic artery was performed, which supplied the VI/VII hepatic tumor. Normal immediate postprocedure single photon emission computed tomography (SPECT)/CT was performed.

A follow-up abdominal CT performed after 2 months showed a sizable residual lesion in the medial aspect of the lesion. The outcome of TARE was not satisfactory; therefore, the patient underwent an extra session of transarterial chemoembolization (TACE), which was performed via the transarterial injection of 50 mg of doxorubicin hydrochloride loaded into 2 ml of DC beads (biocompatible).

The patient continued regular postoperative follow-up examinations. Four months later, a new lesion was found and treated with one session of microwave ablation, which was performed under and CT-guided insertion under general anesthesia. The microwave antenna was moved into the lesion in segment VIII, and a 100-W generator set was used to deliver maximum power with the automatic impedance control method.

Six months postmicrowave ablation, the patient attended the hospital with history shortness of breath, productive cough, and chest pain. On examination, vital signs were normal. A bronchoscopy was performed and was reported as normal uneventful procedure.

**Fig. 2:**
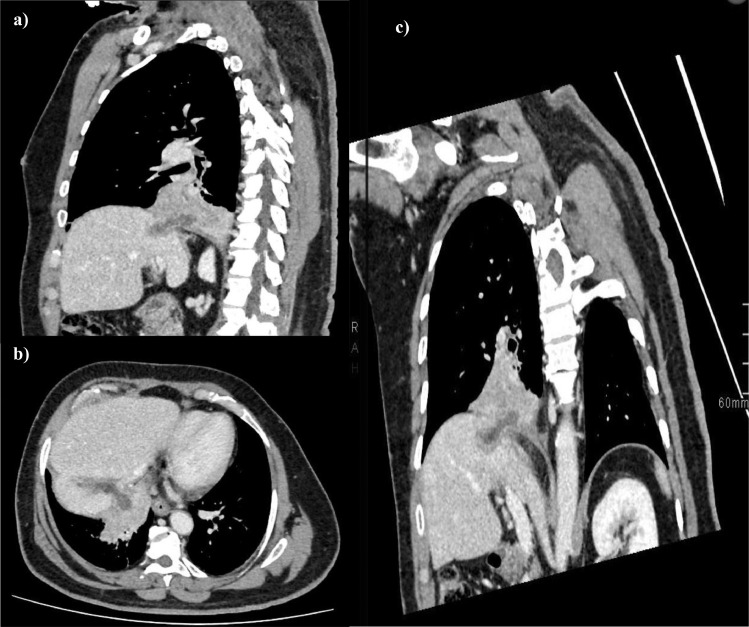
Biliopleural fistula post-RFA. Enhanced CT chest sagittal (A), axial (B), and coronal oblique reformat (C). There is fluid attenuation tract extends from the liver dome to the right lower pleural cavity with secondary right lower lobe mass-like consolidation.

**Fig. 3:**
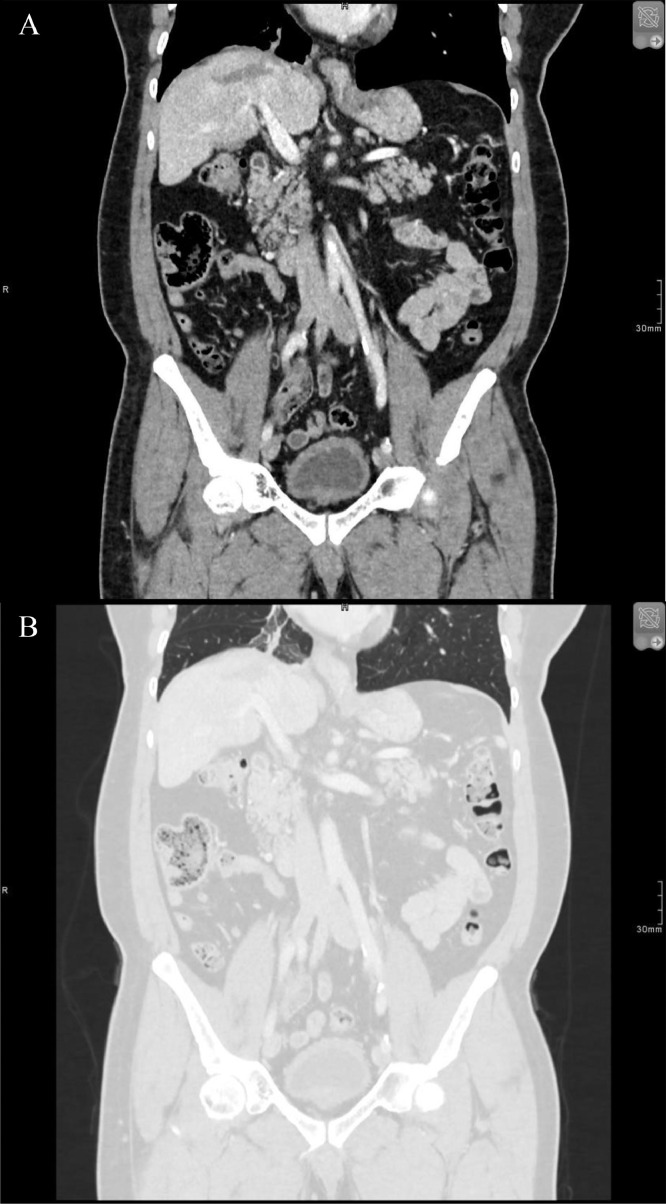
Follow-up enhanced CT abdomen. Coronal soft tissue (A) and lung window (B) images. Regression of the pleural components of the fistula tract and the right lower lobe consolidation with small residual atelectasis and ground-glass opacity.

Chest and abdomen CTs were acquired following administration of intravenous contrast. The thoracic CT scan demonstrated hepatic biloma at the ablation site with cranial extension into the right lower pleural space with right anterior basal mass-like consolidation (Figs. 2 and 3). This fistulous communication between the superior part of the ablated hepatic zone and the adjacent lower pleural cavity raised the suspicion of TBF. Laboratory results revealed abnormalities (ESR 37 mm/h (reference 0-10 mm/h); AST 30U/L (reference 8-48 U/L); ALT 33 U/L (reference 12-78 U/L); TB, fungal, and sputum cultures were all negative).

**Fig. 4:**
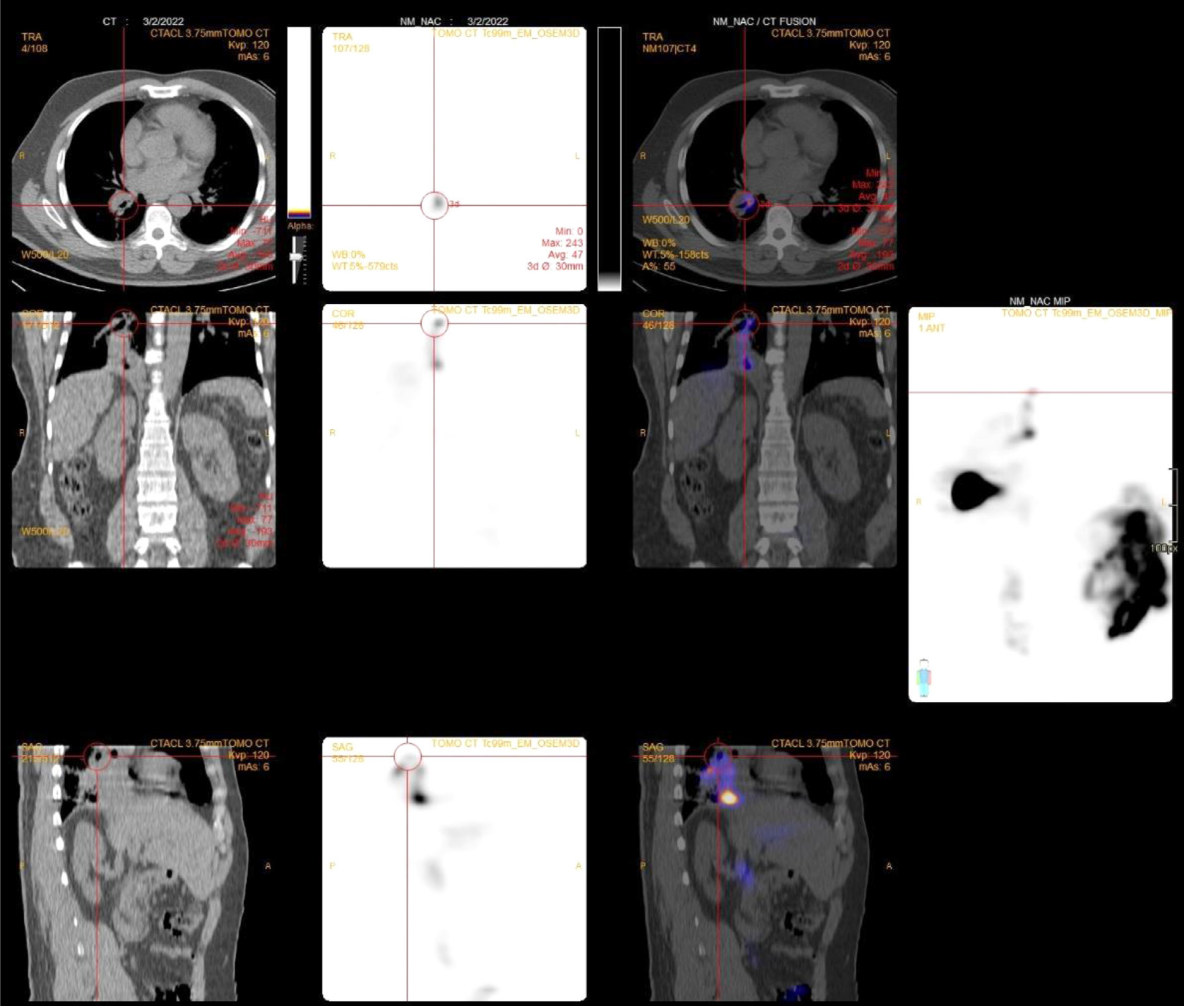
Technetium-99m (Tc-99m) HIDA scan and SPECT/CT scan after 1 hour after injection of radiotracer show tracer passage cranial to the right lower lung, findings confirmed the biliary uptake.

In order to establish the presumed diagnosis of TBF, a technetium-99m (Tc-99m) HIDA scan was performed. Initial dynamic images of the abdomen were performed immediately after intravenous injection of 185MBq of Tc99m-HIDA over 55 minutes, showing prompt hepatic uptake followed by excretion of the tracer into the bile duct and bowel within 25 minutes of radiotracer injection. A delay planer and SPECT/CT scan performed at 1 and 2 hours postradiotracer injection revealed tracer passage cranial to the right lower lung (Fig. 4). This confirmed the diagnosis of pleurobiliary fistula (PBF). The subsequent follow-up abdomen CT revealed no residual or recurrent tumors. The patient was treated conservatively with significant symptom improvement.

## Discussion

Using microwave ablation to treat both primary and secondary liver tumors in a timely manner has been proven an effective therapy, because of its quick ablation duration and steady intratumoral temperature, and the ability to treat large lesions [2]. This is a relatively safe procedure, but it may result in a variety of consequences, such as vascular injury, biliary damage, and infection. TBF is extremely uncommon [3]. The prevalence of biliary tract thermal ablation complications (such as biliary strictures, bilomas, and bile leaks) varies from 0.1% to 12% [4]. The TBF can connect to the bronchi (also called BBF)or the pleural space (also called a PBF) . Tc-99m HIDA radiopharmaceutical is a hepatobiliary agent that is extracted and excreted by hepatocytes using the same cellular mechanism as bilirubin and bile; however, unlike bilirubin, it is not conjugated. The radiopharmaceutical then follows the course of bile through the biliary system and into the bowel.

Cholescintigraphy is a safe, easy, and inexpensive way to confirm that there is communication between the biliary tree and a bile-containing collection or fistulous tract. The SPECT/CT is a hybrid study that combines SPECT and CT scans. By putting these tests together, the technology gives a full and clear picture of the body's anatomy and physiology. The primary objective of this tomographic imaging system is to offer a better 3D image of the radioactive distribution within the patient, improving contrast and image quality.

Our patient exhibited a progressive accumulation of radiotracer in delay images with normal early TC-99m HIDA images. These emphases the importance of delay images in suspected fistula cases. If tracer accumulates in the pleural cavity, the likelihood of biliopleural fistula is more than biliobonchial fistula, in which tracer accumulation takes place in the bronchial tree. The negative bronchoscopy results with no bile found in the lavage fluid were consistent with the biliopleural fistulous type [5,6].

Any event that damages the biliary tract, diaphragm, and/or bronchioles might lead to the formation of a TBF. A TBF can also be caused by a local infection, a blocked biliary tract, a tumor, an injury, surgery, or procedures like thermal ablation or TACE [7].

Peacock reported the first instance of TBF in 1850, which was caused by a local infection [8]. The patient had undergone various interventional procedures, including Y-90 therapy, TACE, and the MWA with thermal impact, which resulted in the development of a biloma and aggravated the progression of PBF. Thermal damage to the diaphragm, local infection following ablation with abscess development, biliary stricture, or biloma formation are all probable explanations of TBF [7].

There have been roughly 16 articles published on TBF following thermal ablation therapy since 2002, according to a literature analysis [3,4,7,[9], [10], [11], [12], [13], [14], [15], [16], [17], [18], [19], [20], [21]]. The age of these patients ranged from 38 to 76 years. Biliptysis, coughing bile, was the most characteristic symptom of Thorachobiliary fistulaTBFCough, chest pain, fever, jaundice, and abdominal pain were additional symptoms [9]. Large lesions and lesions in the right hepatic lobe, segments VII and VIII, have a greater risk of PBF than those in other segments. Their closeness to the diaphragm, with less than 3 mm separating the lesion from the diaphragm, also contributes to these consequences [4]. The occurrence of TBF following thermal ablation ranged from 5 days to 17 months. Biliary neoplasia, biliary tract infections, and biliary obstructions were all risk factors that influenced the onset of symptoms [10]. Other methods, such as percutaneous transhepatic cholangiography, endoscopic retrograde cholangiopancreatography, CT, or magnetic resonance cholangiopancreatography might be helpful for diagnosis. Similarly, 3 of the 16 reported cases are complications of Microwave ablation, while the others are complications of radiofrequency ablation [3,4,18].

CT scan is usually the initial imaging modality, and even though it may not depict the fistula tract directly, it can disclose indirect abnormalities such as pleural effusion, lung consolidation, subphrenic fluid collection, hepatic biloma, or abscess.

In the presented case, pneumonia or lung neoplasm can be differential for the right lower lobe mass-like opacity. However, constellation of HIDA scan findings and improvement of lung consolidation following therapy confirmed the diagnosis [7,11].

## Conclusion

This case showed a rare thermal ablation complication with extrahepatic thermal damage. Patients who have had thermal ablation treatment, especially those with large tumors that are close to the diaphragm, need to be watched closely to prevent problems with the biliary system or damage to the diaphragm.

## Human rights statement

All procedures followed were in accordance with the ethical standards of the responsible committee on human experimentation (institutional and national) and with the Helsinki Declaration of 1964 and later versions.

## Patient consent

Informed consent was obtained from the patient for the publication of this case report.
